# In Vitro Evidence for the Efficacy of Manuka Honey and Its Components Against the Major Human Pathogenic *Sporothrix* Species

**DOI:** 10.3390/ph18040534

**Published:** 2025-04-06

**Authors:** Andrea Reis Bernardes-Engemann, Fernando Almeida-Silva, Levi G. Cleare, Jefferson D. da Cruz, Jefferson Rocha de A. Silva, Walter Sotto M. Fernandes Neto, Rosely Maria Zancopé-Oliveira, Ana Claudia Fernandes Amaral, Joshua D. Nosanchuk, Rodrigo Almeida-Paes

**Affiliations:** 1 Fundação Oswaldo Cruz, Instituto Nacional de Infectologia Evandro Chagas, Laboratório de Micologia, Rio de Janeiro 21040-900, RJ, Brazil; andrea.engemann@ini.fiocruz.br (A.R.B.-E.); fernan-do.almeida@ini.fiocruz.br (F.A.-S.); rosely.zancope@ini.fiocruz.br (R.M.Z.-O.); 2 Albert Einstein College of Medicine, Departments of Medicine (Division of Infectious Diseases) & Microbiology & Immunology, Bronx, NY 10461, USA; levi.cleare@einsteinmed.org (L.G.C.); josh.nosanchuk@einsteinmed.edu (J.D.N.); 3 Fundação Oswaldo Cruz, Instituto de Tecnologia em Fármacos (Farmanguinhos), Laboratório de Química de Produtos Naturais, Rio de Janeiro 21040-900, RJ, Brazil; jefferson_dacruz@hotmail.com; 4 Universidade Federal do Amazonas, Instituto de Ciências Exatas, Departamento de Química, Laboratório de Cromatografia, Manaus 69077-000, AM, Brazil; jrocha_01@yahoo.com.br (J.R.d.A.S.); wssottoo@gmail.com (W.S.M.F.N.)

**Keywords:** antifungal activity, honey, hydrogen peroxide, sporotrichosis, treatment

## Abstract

**Background/Objectives**: While various clinical manifestations occur in sporotrichosis, cutaneous forms predominate. The recommended sporotrichosis treatment is itraconazole, an antifungal with certain restrictions. In recent years, the observation of reduced treatment effectiveness in some patients has arisen, possibly due to *Sporothrix* spp. resistance mechanisms. Consequently, there is a growing need for alternative therapeutic approaches. This study investigates the antifungal activity of manuka honey (MH) against pathogenic species of the genus *Sporothrix*. **Methods**: In this study, we assessed MH antifungal efficacy across concentrations ranging from 5% to 40% against 26 *Sporothrix* spp. isolates. In addition, its components were evaluated through chromatography and other in vitro techniques. **Results**: Minimum inhibitory concentrations of MH were found to be 15–40%, 10–15%, and 5–10% for *Sporothrix brasiliensis*, *Sporothrix schenckii*, and *Sporothrix globosa*, respectively. Purified methylglyoxal did not hinder *Sporothrix* growth. The MH antifungal potential was compromised through treatment with catalase or filtration through a 0.22 µm cellulose membrane. Chromatographic analysis of the volatile organic compounds (VOCs) present in MH identified 40 VOCs, including carbonyl compounds, alcohols, esters, aromatic hydrocarbons, heterocyclic compounds, terpenoids, and carboxylic acids. Additionally, two phenolic compounds were identified as potential markers for the authentication of MH, along with a disaccharide that may contribute to its antifungal activity. **Conclusions**: MH has demonstrated biological activity against the most significant *Sporothrix* species with pathogenic impact on humans. This suggests its consideration in future research endeavors focused on novel topical treatments for cutaneous sporotrichosis in both human and animal subjects.

## 1. Introduction

The medicinal use of honey is well documented in ancient records, spanning the globe [1]. Extensive research has been conducted on its antimicrobial attributes, whose effectiveness varies based on factors such as geographical origin, plant source, bee traits, environmental factors, processing techniques, and storage conditions [2]. The bactericidal capacity of honey is attributed to its high osmolarity, specificity, low water activity, production of hydrogen peroxide, and the existence of various other phytochemical constituents [1,3,4].

In particular, among various honey types, the medical-grade manuka honey (MH) stands out as one of the main and most scientifically investigated honey types [5]. Renowned for its formidable bactericidal properties, MH presently finds clinical utility in the treatment of infected wounds [4,6]. This honey type is derived from nectar collected by *Apis mellifera* bees from the Manuka bush (*Leptospermum scoparium*) native to New Zealand and Australia [7]. The constitution of MH encompasses carbohydrates, proteins, minerals, fatty acids, phenolic substances and flavonoids [5]. While these constituents are prevalent in other honey types, MH boasts distinctive attributes, notably an exceptionally elevated level of methylglyoxal (MGO), a compound closely linked to the MH antibacterial efficacy [8]. Additionally, MH contains methyl syringate glycoside (leptosperin), a unique presence that potentially serves as an authentication marker for MH [9].

The antimicrobial activity of MH is intricately linked with the unique manuka factor (UMF), a parameter tied to the concentrations of MGO and total phenols contents. A grading system was developed to categorize the strength of MH based on UMF, spanning from 5+ to 25+ UMF [10]. Beyond its direct antibacterial properties, UMF-endowed honey displays the additional ability to activate macrophages, resulting in the release of TNF-α, IL-1β, and IL-6. These cytokines play pivotal roles in combatting microbial infections and facilitating tissue recuperation [5].

The recognition of the robust efficacy of MH led to the development of medical-grade honey, which has since found a multitude of applications within the clinical setting. The main formulations of medical-grade honey are as a topical ointment and in impregnated-dressings for the treatment of superficial wounds and burns [6,11]. Both formulations promote wound healing; however, more importantly, they also prevent and treat microbial infections, especially those caused by multidrug-resistant microorganisms [12].

Sporotrichosis is a subcutaneous mycosis caused by thermodimorphic fungi of the genus *Sporothrix*. While it predominantly manifests in the lymphocutaneous form, confined to the skin and subcutaneous tissue, instances of extracutaneous and atypical presentations also occur [13]. It is a cosmopolitan infection, and, in some countries, such as Brazil, Mexico, India, and China, it is considered endemic [14,15]. Notably, a significant facet is the emergence of animal sporotrichosis, particularly among domestic cats that can transmit the disease to humans, thereby contributing to its propagation [16]. For instance, within this zoonotic scenario, sporotrichosis has progressively disseminated from southeastern Brazil to encompass the entire country over the last two decades [17]. For the treatment of human sporotrichosis, itraconazole is used as the drug of choice according to international protocols. This antifungal agent is especially indicated for treating cutaneous forms of the disease, which constitute over 95% of cases [13]. However, apart from concerns related to nephrotoxicity [18], itraconazole is contraindicated for pregnant women due to its potential teratogenic effects [19]. Additionally, a growing number of cases involving itraconazole refractory sporotrichosis in humans and other animals have surfaced in recent years [20,21,22].

In an attempt to find innovative therapeutic avenues targeting cutaneous sporotrichosis, we undertook an examination of manuka honey’s potential antifungal attributes against the primary species responsible for sporotrichosis in both human and animal hosts. Furthermore, we delved into the constituents of MH that might contribute to this observed antifungal efficacy.

## 2. Results

### 2.1. Manuka Honey Has Antifungal Activity Against Sporothrix spp.

Three reference *Sporothrix* strains of the major human pathogenic species (*Sporothrix brasiliensis*, *Sporothrix globosa*, and *Sporothrix schenckii*) were tested against two types of honey: two MHs with different UMFs. Table 1 presents the minimal inhibitory concentration (MIC) values for the two MH samples tested. The MH UMF 15+ had higher efficacy than the MH UMF 5+ against the yeast form of the three *Sporothrix* species tested. As the MH UMF 15+ had higher antifungal activity, it was used in the further experiments and chromatographic analyses. The two non-manuka honey samples had no discernable effect against all major human *Sporothrix* pathogenic species, with MICs higher than 40% *v*/*v*.

### 2.2. Manuka Honey Is Active Against the Two Morphotypes of Sporothrix spp.

Table 2 shows the activity of MH UMF 15+, filtered and unfiltered, against the mycelial and yeast forms of the reference strains of *Sporothrix* spp. The MIC for *S. brasiliensis* and *S. schenckii* was slightly higher for the mycelial form of the fungus, while for *S. globosa*, it was higher for the yeast form. It was also observed that filtration decreased the anti-*Sporothrix* activity of MH UMF 15+ for both morphotypes.

### 2.3. The Antifungal Activity of the Manuka Honey Against Sporothrix spp. Is Stable

The in vitro antifungal activity tests described above were repeated two years later using the same batch and before the expiration date of the MH UMF 15+. The same results as those shown in Table 2 were observed.

### 2.4. Manuka Honey Is Active Against Non-Wild Type Sporothrix spp.

This analysis included 11 *S. brasiliensis* strains exhibiting a non-wild-type profile for at least one traditional antifungal drug, to evaluate the effectiveness of MH against these strains. Table 3 details their antifungal susceptibility profile, along with the description of specific antifungal drugs to which they exhibit reduced susceptibility.

**Figure 1:**
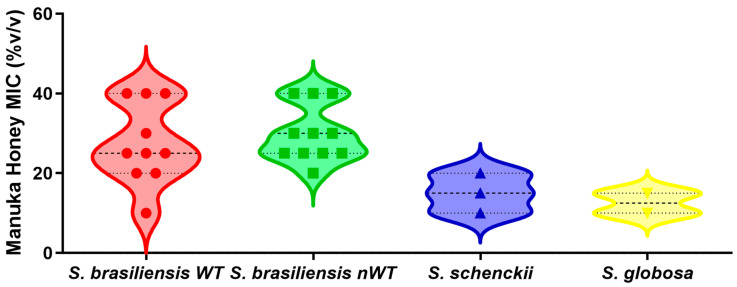
Minimal inhibitory concentrations (MIC) of manuka honey UMF15+ against three clinically relevant *Sporothrix* species. The species *S. brasiliensis* is further categorized in two groups: wild-type (WT) and non-wild-type (nWT) strains, based on their susceptibility to conventional antifungal drugs. The violin plots depict the distribution, central tendency, and variability of the minimal inhibitory concentration of the manuka honey. Each symbol within the violins represents the individual MIC values for each strain. The dashed line inside violins indicates the median MIC, while the dotted lines represent the interquartile range. Statistical analysis (Kruskal–Wallis test followed by the Dunn’s multiple comparison test) revealed no significant differences among the MIC values of the four groups studied.

Figure 1 shows the MIC values of MH UMF 15+ against the yeast form of 26 *Sporothrix* spp. strains. The MICs of MH were found to be within the following ranges for *S. brasiliensis*, *S. schenckii*, and *S. globosa*, respectively: 15–40% *v*/*v*, 10–15% *v*/*v*, and 5–10% *v*/*v*. Four strains had a non-wild-type phenotype for more than one antifungal drug. According to the Kruskal–Wallis test followed by the Dunn’s multiple comparison test, there was no significant difference between the MICs for wild-type and non-wild-type *S. brasiliensis* strains (*p* > 0.05).

### 2.5. Manuka Honey Effects on Sporothrix spp.

**Figure 2:**
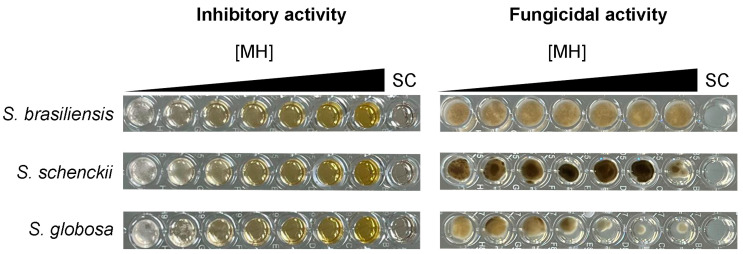
Manuka honey inhibits, but does not kill, *Sporothrix* spp. The left panels display the results of the inhibitory activity assay conducted with increasing concentrations of manuka honey ([MH]): 0%, 5%, 10%, 15%, 20%, 30%, and 40% *v*/*v*, tested against three reference strains. The right panels show the outcomes of the fungicidal activity assay, where a 5 µL aliquot from each well of the inhibitory assay was plated onto potato dextrose agar. Notably, all strains were able to grow at the highest manuka honey concentration tested, despite the inhibition observed. For all experiments, a sterility control (SC) was included, with no fungal inoculation. Images are representative of triplicate experiments.

The mechanism of action of MH against *Sporothrix* spp. is likely to be fungistatic, as the minimum fungicidal concentration of this honey type against all strains herein studied was higher than 40% *v*/*v*, meaning that MH only inhibits the growth of *Sporothrix* spp., as depicted in Figure 2. Furthermore, we evaluated the effects of some components of the MH against *Sporothrix* spp. The purified methylglyoxal standard showed no activity against the tested *Sporothrix* strains, with MIC values exceeding 2048 µg/mL for all strains. The treatment of MH with catalase eliminated its antifungal activity, resulting in MIC values greater than 40% *v*/*v* for all strains.

### 2.6. Bioactive Manuka Honey Composition

**Figure 3:**
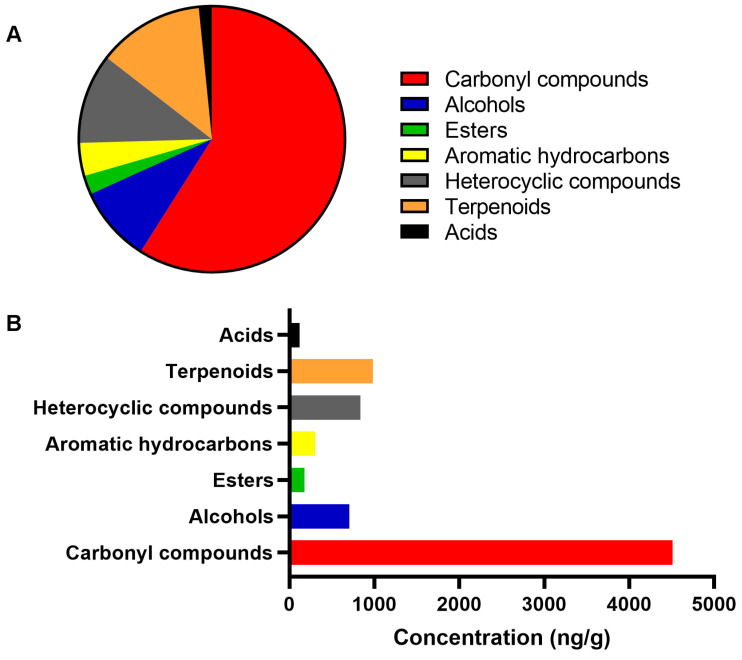
Volatile composition of the manuka honey. The graphs depict the percentage of each chemical class of compounds in the sample evaluated by gas chromatograph coupled to mass spectrometry (**A**) and their concentration, in ng/g (**B**).

The results obtained by solid phase microextraction (SPME)/headspace indicated the presence of 40 volatile organic compounds (VOCs), 15 of which were carbonyl compounds, 4 alcohols, 2 esters, 2 aromatic hydrocarbons, 8 heterocyclic compounds, 7 terpenoids, and 2 carboxylic acids (Figure 3). Table S1 lists all VOCs identified in the MH UMF 15+ sample evaluated. The predominant class of volatile constituents in MH were carbonyl compounds, representing 59.0% of the total substances identified, with aldehyde, nonanal (1112.35 ppb) being the compound with the highest concentration detected in the aroma. The second most abundant class were terpenoids (12.9%), while heterocyclic compounds represented the third largest class, representing 11.0% of the total substances identified in the MH aroma (Figure 3). In addition, the results related to the odor activity value (OAV) of 23 compounds are also listed in Table S1. The chemical markers that characterize MH, methylglyoxal (293.5 ppb), 2′-methoxyacetophenone (776.2 ppb), and 2-methylbenzofuran (76.80 ppb), were present in the honey aroma. Among all the compounds found in MH, nonanal showed the highest odorous activity (OAV = 1112.3), contributing to the perception of aromatic notes that refer to orange. Other compounds that impact the MH aroma with OAV > 50 resemble sweet, flowery due to lilac aldehyde B (OAV = 419.3); citrus, fruity, sweet–waxy, decanal contribution (OAV = 104.3); menthol (OAV = 134.8), and fresh, floral, rose, green, descriptive notes of the aroma of geranylacetone (OAV = 93.7). The study also provides results regarding the LC-MS chromatographic profiles of the MH UMF 15+. The main compounds identified in the honey in the negative ionization mode were a disaccharide, 4-O-beta-D-gentiobiose, and two phenolic compounds, leptosperin (methyl syringate 4-O-beta-D-gentiobiose), and pinobanksin-3-O-pentenoate.

## 3. Discussion

Honey has been used as wound dressing for centuries, but the basis for the efficacy of honey has only recently been established [23]. Honey has multiple bioactivities that accelerate the healing process. For example, its acidic pH increases the release of oxygen from hemoglobin, making the wound environment less favorable for the activity of destructive proteases. Additionally, the high osmolarity of honey draws fluid from the wound bed to create an outflow of lymph, similar to negative pressure wound therapy [24]. These properties would be attractive in the context of cutaneous sporotrichosis. Therefore, the determination of possible anti-*Sporothrix* activity of some honey types is an interesting area of research for developing new therapeutic approaches to fight this disease.

The antimicrobial properties of MH have been extensively studied [5,7,25,26], but to the best of our knowledge, this is the first report of its anti-*Sporothrix* activity. In the present study, MH was effective against the three major species that cause sporotrichosis, while two Brazilian honey types were not. This suggests that the anti-*Sporothrix* activity is dependent on the honey type and on the UMF rating of the MH. In this study, the MH UMF 15+ was more effective than MH UMF 5+ against *Sporothrix* spp. This is different from what has been observed for bacteria [5], suggesting distinct mechanisms of action. Our results showed that MH is fungistatic against *Sporothrix* spp., which means that it inhibits the growth of the fungus but does not kill it. This is similar to the effect of the major antifungal drug recommended for sporotrichosis treatment, itraconazole, which is a fungistatic compound [27]. The ability of MH to inhibit *Sporothrix* growth coupled to its ability to enhance microbial sensing by T cells [28] and to mediate pro-inflammatory cytokine release by macrophages [5] makes MH an interesting therapeutic compound for sporotrichosis. Since MH is effective against both mycelial and yeast forms of *Sporothrix* spp., it has both prophylactic and therapeutic potential against these species.

The emergence of *Sporothrix* strains presenting high MIC values for the actually prescribed antifungal drugs for sporotrichosis, known as non-wild-type *Sporothrix* strains [29,30], is a worrying event. The infections caused by these antifungal drug-resistant strains are usually difficult to treat, requiring high antifungal (itraconazole, terbinafine, or amphotericin B) doses and prolonged treatment durations [20,31]. MH is active against certain multi-drug resistant bacteria [5,12,32], and our results showed that this honey type is also effective against non-wild-type *S. brasiliensis* strains, which is further interesting in vitro evidence supporting its use for sporotrichosis treatment.

The antimicrobial properties of various honey types have been attributed to several physical and chemical properties such as acid pH, osmotic effect, hydrogen peroxide release when hydrated, and presence of certain phenolic compounds [26]. For MH, its high MGO concentrations is an additional and important antimicrobial mechanism [5]. Pathogenic *Sporothrix* strains can grow in a broad pH range, from 2.4 to 12, depending on the morphotype, and are also tolerant to high osmotic pressure [33], so the MH pH or osmotic properties are not likely relevant in its anti-*Sporothrix* action.

The VOCs present in honey originate from a complex network that involves both the plant and the bees. However, even considering this intricate biosynthetic mechanism, a set of substances generated during this process are characterized as MH markers, differentiating it from other honey types, e.g., from kanuka honey and jelly bush honey [34,35]. Additionally, depending on the concentration of these markers, it is possible to identify the geographical location where they were produced [36,37].

In the present work, the volatile compounds detected in MH include, in addition to methylglyoxal, a compound referenced by non-peroxide antibacterial activity [35] and other bioactive compounds. For example, nonanal and decanal demonstrated antifungal activity on *Aspergillus flavus* [38], while linalool oxide and its isomers showed high biological activity against *Penicillium citrinum*, *Rhizopus oryzae,* and *Chaetomium globosum* [39]. Additionally, menthol and cyclic monoterpene alcohol have antimicrobial, anticancer, and anti-inflammatory activities [40], and have recently been evaluated alone or in combination with itraconazole, voriconazole, and amphotericin B on the growth inhibition and biofilm formation of *Candida albicans* [41].

In addition, methyl syringate was detected in Moroccan Zantaz honey, which showed antioxidant, immunomodulatory, and antiproliferative properties in assays with Caco-2 and THP-1 cells. Methyl syringate has also been identified in manuka, kanuka, and asphodel honeys, and smaller amounts have been detected in honeys derived from other species [42]. Concerning the 2,3-dihydro-3,5-dihydroxy-6-methyl-4H-pyran-4-one, this compound may be a product generated from the Maillard reaction, and shows important antioxidant activities in assays with 2,2-diphenyl-1-picrylhydrazyl, 2,2′-azino-bis(3-ethylbenzothiazoline-6-sulfonic acid) or ferric ions [43].

In our work, the mass spectra analysis of chromatograms, acquired through LC-QTOF-HRMS/MS with electrospray ionization (ESI), facilitated the identification of three main compounds, 4-O-beta-D-gentiobiose, leptosperin, a methyl syringate derivative, and pinobanksin-3-O-pentenoate within MH. Notably, all these compounds had been previously isolated and identified in other MH [44]. The preference for the QTOF mass analyzer in screening was primarily driven by its exact mass determination capability. The primary component, the disaccharide 4-O-beta-D-gentiobiose, exhibited a fragment [M-H]-1 at *m*/*z* 341.1100. This sugar was identified as a noteworthy constituent in the MH sample and is potentially linked to in vitro antibacterial activity against *Helicobacter pylori* [45]. The phenolic compounds, leptosperin and pinobanksin-3-O-pentenoate, exhibit distinctive MS fragments at *m*/*z* 581.1722 and 353.0987, respectively. They are recognized as chemical markers for MH authentication and are linked to various pharmacological activities associated with this honey [44,46].

The assay with the methylglyoxal standard showed that this molecule does not have anti-*Sporothrix* activity, at least singly, as occurs for some bacteria, such as *Pseudomonas aerugionosa* [47]. This does not mean that methylglyoxal does not have a role in the antifungal activity of the MH, because it may act synergistically with other honey components. Despite this result, the identification of phenolic compounds, including methyl syringate and its derivatives, along with the identification of a disaccharide and other significant chemical markers, constitutes a complex array of compounds whose antimicrobial activity has been previously confirmed. Further, the identification of phenolic compounds such as methyl syringate and derivatives, disaccharide, and other important chemicals is part of the complex of compounds whose antimicrobial activity has already been verified.

The detection of phenolic compounds, including methyl syringate and its derivatives, together with the identification of a disaccharide and other significant chemical markers, constitutes a complex set of compounds whose antimicrobial activity has been previously confirmed and may be associated with the observed anti-*Sporothrix* activity. Hydrogen peroxide is implicated in the anti-*Sporothrix* action of the MH, since catalase treatment of the honey abolished antifungal activity. *Sporothrix* spp. are highly sensitive to hydrogen peroxide [48,49], which may explain the fungal growth inhibition.

## 4. Materials and Methods

### 4.1. Honey Samples

Two samples of MH (Kiva Health, Honolulu, HI, USA) with different UMF grades (5+ and 15+) were studied. The MH samples have manuka UMF certifications. Additionally, two Brazilian honey samples (Amigos da Terra, Nova Friburgo, RJ, Brazil) were tested. These Brazilian samples comprised honey derived from *Citrus sinensis* and a composite of flowers from various plants, predominantly *Eucalyptus* spp. The Brazilian honey samples adhered to Good Manufacturing Practices and the General Principles of Food Hygiene during their production. To ensure optimal conditions, all honey samples were stored in a lightless environment at room temperature, maintaining a dry atmospheric setting.

### 4.2. Fungal Strains

The reference strains *Sporothrix brasiliensis* CFP 00722, *Sporothrix schenckii* CFP 00448, and *Sporothrix globosa* CFP 01021 were used throughout the study. In addition, an additional set of 23 clinical strains was studied, including 2 *S. schenckii*, 1 *S. globosa*, and 20 *S. brasiliensis*. Notably, the *S. brasiliensis* strains were previously classified as either wild-type (*n* = 10) or non-wild-type (*n* = 11) to at least one antifungal drug commonly employed in treating sporotrichosis [29,31].

### 4.3. Culture Conditions

The *Sporothrix* spp. strains were cultivated on either potato dextrose agar or brain–heart infusion agar (both from Becton-Dickinson and Company, Sparks, MD, USA) for seven days at 25 °C or 35 °C to obtain conidia or yeast cells, respectively [48]. Post-cultivation, the cells were suspended in phosphate-buffered saline (Sigma-Aldrich, St. Louis, MO, USA) in a concentration of 1 × 10^6^ cells/mL, after counting on a Newbauer chamber. This suspension was used in the following experiments.

### 4.4. Antifungal Activity of Honey Samples

The four honey samples were diluted using an RPMI 1640 medium (Sigma-Aldrich) at 40% *v*/*v*, 30% *v*/*v*, 25% *v*/*v*, 20% *v*/*v*, 15% *v*/*v*, 10% *v*/*v*, and 5% *v*/*v* final concentrations. These samples were tested in sterile 96-well plates against the three *Sporothrix* reference strains, with each test conducted in triplicate. Final concentrations of yeast or conidia in test conditions were 5 × 10^4^ cells/mL. Plates were incubated at 35 °C for 48–72 h, in a condition similar to that used internationally for antifungal susceptibility testing of *Sporothrix* species [30]. Negative controls included wells with only RPMI 1640 medium or honey samples. Fungal growth control wells consisted in *Sporothrix* cells suspended in RPMI 1640 medium at the abovementioned concentration, without any honey sample. The minimal inhibitory concentration (MIC) of each honey sample was determined as the lowest honey concentration that visibly inhibited 100% fungal growth. The clinical strains underwent similar testing, but exclusively using the honey sample that demonstrated superior inhibition outcomes with the reference strains. This particular honey sample was also used in subsequent experiments of this study. Additionally, to ascertain whether the antifungal activity was fungistatic or fungicidal, subcultures were prepared by transferring 5 µL of wells that showed no fungal growth onto potato dextrose agar. After incubating these subcultures for seven days at 25 °C, the growth of *Sporothrix* colonies was assessed.

### 4.5. Filtration Impact on the Honey Antifungal Activity

To evaluate the influence of filtration on the antifungal efficacy of honey, we replicated the MIC experiment, as outlined earlier. However, in this instance, we employed filtered honey in lieu of raw honey. The filtration process involved using a polystyrene 0.22 µm syringe filter, after which the filtered honey was subjected to the same dilutions (ranging from 40% *v*/*v* to 5% *v*/*v*) that were utilized in the prior testing. The aim was to compare the MIC values of both crude and filtered honey.

### 4.6. MGO Activity Against Sporothrix spp.

The antifungal activity of MGO against the reference *Sporothrix* strains was evaluated using the same protocol described for antifungal susceptibility testing of pathogenic *Sporothrix* species [30]. The primary alteration involved substituting antifungal drugs with purified MGO (Sigma-Aldrich). In the test conditions, the MGO concentrations spanned from 2048 to 4 µg/mL, aligning with previously documented parameters [47].

### 4.7. Catalase Influence on the Honey Antifungal Activity

To assess the potential impact of catalase, we conducted a replication of the minimal inhibitory concentration experiment outlined earlier. However, in this iteration, we introduced catalase from Sigma-Aldrich into each honey dilution, maintaining a final enzyme concentration of 0.2% *w*/*v*, as detailed previously [50]. Within this setup, negative control wells received only RPMI 1640 medium and catalase, while the growth control wells contained *Sporothrix* cells, the culture medium, and catalase at a concentration of 0.2% *w*/*v*. The MIC values of the honey in the presence and absence of catalase were compared.

### 4.8. Honey Composition Analysis

#### 4.8.1. Sample Preparation and Extraction

Fifty milligrams of MH were added to 0.75 g NaCl, 5 mL Milli-Q water (Millipore, Burlington, MA, USA) and 3-octanol (internal standard, 10 mg/L standard solution in absolute ethanol) in a 20 mL vial. The flask was sealed with a cap aluminum septum and placed in a water bath at 40 °C, under magnetic stirring. After the equilibration time (10 min), a Supelco SPME device with a fiber coated with 65 µm polydimethylsiloxane/divinylbenzene (Bellefonte, PA, USA) was inserted into the vial containing the sample. The fiber was exposed to the headspace for 40 min at 40 °C. Once sampling was finished, the fiber was withdrawn into the needle and transferred to the injection port of the gas chromatograph/mass spectrometer (GC–MS) system for 4 min at 260 °C to desorb the analytes in splitless mode.

#### 4.8.2. Volatile Compounds Analysis by Gas Chromatography Mass Spectrometry

The analysis of volatile compounds via GC–MS was conducted using a Shimadzu GC-2010 plus gas chromatograph (Shimadzu, Duisburg, Germany) coupled with a QP-2010 Mass Selective Detector (Shimadzu), ionization voltage 70 eV. The system was equipped with a nonpolar DB-5MS capillary column (Agilent Technologies, Santa Clara, CA, USA) with the following characteristics: 30 m × 0.25 mm, film thickness 0.25 μm, and helium was employed as the carrier gas at a flow rate of 1.0 mL/min. The oven temperature was programmed to increase from 50 °C to 260 °C at a rate of 7 °C/min, followed by an isothermal hold at 260 °C for 5 min. Injector and detector temperatures were maintained at 250 °C. The linear velocity was set at 14 cm/s. The MS interface temperature was 280 °C, with a mass range of 40–700 Daltons, a scan speed of 150 u/s, and an interval of 0.50 s (2 Hz). All analyses were conducted in triplicate. The identification of volatile constituents involved comparing their retention indices (calculated for all volatile constituents using an n-alkane homologous series) and mass spectra with those reported in the literature or available in the Wiley data system library of the GC–MS equipment.

#### 4.8.3. Identification of the Compounds

The experimental conditions were as follows: mobile phase consisting of (A) H_2_O (with 0.1% formic acid) and (B) CH_3_CN, employing a Kinetex column (150 × 4.6, 5 µm) and a gradient profile as follows: 0 min/25% B, 10 min/28% B, 12 min/45% B, 14 min/50% B, 20 min/50% B, 20.1 min/25% B, 25 min/25% B. The temperature was set at 30 °C, with a backpressure of 160 bar. The injection volume was 5 μL, and the flow rate was maintained at 3 mL/min with a split flow rate of 0.3 mL/min. Detection was performed in scan mode using ESI negative ionization. The QTOF Compact Bruker mass spectrometer (Bruker Corporation, Billerica, MA, USA) was employed, operating in the mass range of 100 to 1000 Daltons, utilizing ESI in negative mode. Sodium formate served as the calibrant. ESI source parameters included nebulizer gas (nitrogen, 5.5 bar), dry gas (nitrogen, 12 L/min) at 220 °C, capillary voltage at 4.5 kV, and quadrupole time-of-flight high-resolution mass spectrometry (QTOF/HRMS/MS). Peak information data were compared with literature and database records for identification.

### 4.9. Statistics

The Graph Pad software version 8.4 was used to compare MIC values between groups using the Kruskal–Wallis test followed by the Dunn’s multiple comparison test. A significance level of 5% was used in the analysis.

## 5. Conclusions

The anti-*Sporothrix* activity observed in MH arises from the array of compounds within this intricate matrix, potentially acting synergistically, individually, or in a network of non-covalent interactions with peptides, proteins, carbohydrates, and other components. This conglomeration or cluster of compounds can effectively engage with the biological targets. Further research is essential to translate these findings into clinical applications for treating individuals with sporotrichosis in endemic regions.

## Figures and Tables

**Table 1:** In vitro activity of two manuka honey samples with different unique manuka factors (5+ and 15+) against the yeast form of three *Sporothrix* species of clinical interest.

Strain	Minimal Inhibitory Concentration	
	MH UMF 5+	MH UMF 15+
*Sporothrix brasiliensis* CFP 00722	10% *v*/*v*	<5% *v*/*v*
*Sporothrix schenckii* CFP 00448	30% *v*/*v*	<5% *v*/*v*
*Sporothrix globosa* CFP 01021	30% *v*/*v*	15% *v*/*v*

MH: manuka honey; UMF: unique manuka factor.

**Table 2:** In vitro activity of the manuka honey (unique manuka factor 15+) against the two morphotypes of three *Sporothrix* species of clinical interest.

Strain	Minimal Inhibitory Concentration			
	Mycelial Morphotype		Yeast Morphotype	
	Non-Filtered MH	Filtered MH	Non-Filtered MH	Filtered MH
*Sporothrix brasiliensis* CFP 00722	20% *v*/*v*	>40% *v*/*v*	<5% *v*/*v*	15% *v*/*v*
*Sporothrix schenckii* CFP 00448	15% *v*/*v*	>40% *v*/*v*	<5% *v*/*v*	10% *v*/*v*
*Sporothrix globosa* CFP 01021	10% *v*/*v*	15% *v*/*v*	15% *v*/*v*	20% *v*/*v*

MH: manuka honey; UMF: unique manuka factor.

**Table 3:** Minimal inhibitory concentration (MIC) and non-wild type phenotypes of 11 *Sporothrix brasiliensis* strains evaluated in this study.

Strain	Antifungal Drug MIC (µg/mL)					nWT Drug
	ITR	AMB	TRB	POS	KET	
52731	8.0	8.0	0.5	8.0	0.5	ITR/AMB/TRB/POS
52814	1.0	8.0	0.12	2.0	0.5	AMB
34180	4.0	8.0	<0.015	0.25	0.25	ITR/AMB
17692	0.25	0.5	<0.015	0.12	2.0	KET
18782	0.25	0.5	<0.015	0.12	2.0	KET
17331	0.25	0.5	0.015	0.12	4.0	KET
25758	0.5	0.5	0.03	0.5	8.0	KET
30030	1.0	0.5	<0.015	0.25	4.0	KET
28606	8.0	0.5	0.015	4.0	4.0	ITR/POS/KET
17608	0.5	8.0	0.12	0.5	4.0	AMB/KET
48605	4.0	0.5	<0.015	0.25	0.12	ITR

MIC: minimal inhibitory concentration; ITR: itraconazole; AMB: amphotericin B; TRB: terbinafine; POS: posaconazole; KET: ketoconazole; nWT: non-wild-type.

## Data Availability

The original contributions presented in this study are included in the article/Supplementary Material. Further inquiries can be directed to the corresponding author.

## Supplementary Materials

The following supporting information can be downloaded at: https://www.mdpi.com/article/10.3390/ph18040534/s1, Table S1: Volatile composition of the manuka honey (ng/g) extracted by HS-SPME using PDMS–DVB fiber, followed by DB-5MS GC column. References [51,52,53,54,55,56,57,58,59,60,61,62,63,64,65,66,67,68,69] are cited in the supplementary materials.

## Abbreviations

The following abbreviations are used in this manuscript:

ESI	Electrospray ionization
GC	Gas chromatography
HRMS	High-resolution mass spectrometry
IL	Interleukin
LC-MS	Liquid chromatography
MGO	Methylglyoxal
MH	Manuka honey
MIC	Minimal inhibitory concentration
MS	Mass spectrometry
nWT	Non-wild-type
OAV	Odor activity value
QTOF	Quadrupole time-of-flight
SC	Sterility control
SPME	Solid-phase microextraction
TNF	Tumor necrosis factor
UMF	Unique manuka factor
VOC	Volatile organic compound
WT	Wild-type
