# Supplementary material for: In Vitro Evidence for the Efficacy of Manuka Honey and Its Components Against the Major Human Pathogenic Sporothrix Species
Source: Pharmaceuticals (Basel). 2025 Apr 6;18(4):534. doi: 10.3390/ph18040534 (PMC12030384; doi:10.3390/ph18040534)
Supplement: Supplementary file 1 [file pharmaceuticals-18-00534-s001.zip › pharmaceuticals-3557710-supplementary.pdf]

Table S1. Volatile composition of the Manuka honey (ng/g) extracted by HS-SPME using PDMS–DVB fiber, followed by DB-5MS GC column.

Compound	RI[a]	RI[b]	Manuka honey (ng/g)	Odor threshold[c] (ppb)	OAV[d]	Odor description[e]	Reference
**Carbonyl compounds**							
Benzaldehyde	920	940	454.60	350.0	1.3	Caramel roasted Green	Paravisini et al. 2015; Wang et al. 2022
methylglyoxal	955	970	293.46				
5-methyl furfural	976	987	18.16	50	0.4	Almond floral	Paravisini et al. 2015; Wang et al. 2022
1,3-dihydroxy-2-Propanone	1035	-	61.54				
6-methyl-5-hepten-2-one	990	991	36.16	50	0.7	Fruity, apple-like, musty	Guo et al. 2021.
Phenylacetaldehyde	1033	1044	162.67	24	6.8	honey-like	Söllner et al. 2009
Nonanal	1100	1102	1112.35	1	1112.3	fatty	Paravisini et al. 2015; Wang et al. 2022
p-Menthone	1141	1148	34.03	476	0.07	minty-refreshing	Čiča et al. 2022
Lilac aldehyde B	1155	1154	167.74	0.4	419.3	sweet, flowery	Kreck & Mosandl 2003
3,4-Dimethylbenzaldehyde	1168	1165	893.18	-	-	-	

Decanal	1206	1203	375.44	3.6	104.3	citrus, fruity, sweet-waxy	Zhu et al. 2022
p-Anisaldehyde	1225	1226	67.46	50	1.3	marzipan, sweet, pungent	v. Gemert 1999
1-p-Menthen-9-al	1234	1234	27.34	-	-	-	
2'-methoxyacetophenone	1267	1285	776.17	3,300	0.2	plastic, chemical, petrol	Piornos et al. 2019
Tridecanal	1505	1510	32.05	10,000	0.003	-	Sun et al. 2014
**Alcohols**							
1,3-Propanediol	809	814	111.78	-	-	-	
Hotrienol	1119	1119	102.30	110	0.9	Fresh, floral, fruity	Pino & Mesa 2006
2-Ethylhexan-1-ol	1025	1028	437.53	13	33.6	sweet, slightly flowery	Song et al. 2021
1-Nonanol	1157	1161	56.00	50	1.1	Honey	Pino 2012
**Esters**							
methyl syringate	1741	1744	131.03	-	-	-	
2-hydroxy-gamma-butyrolactone	2138	2142	44.81	-	-	-	
**Aromatic hydrocarbon**							
4-Methylanisole	1001	1005	69.22	0.01	6.9	Ylang oil and violet- like	Fan et al. 2018
2-Vinylanisole	1009	-	238.48	-	-	-	
**Heterocyclic compound**							
2-Methylbenzofuran	1121	1123	76.80	-	-	-	

2,3-Dihydro-3,5-dihydroxy-6-methyl-4H-pyran-4-one	1126	1130	224.64	-	-	-	
5-Hydroxymethylfurfural	1220	1224	250.82	40	6.3	Chamomile-flower	Lasekan 2013
2(5H)-Furanone	911	913	47.15	-	-	-	
Furan-2,5-dicarboxaldehyde	1071	1076	77.43	-	-	-	
Benzofuran-3-carbaldehyde	1844	-	71.85	-	-	-	
2-hydroxy-3-methyl-4H-pyran-4-one	2027	2030	35.72	-	-	-	
2H-Pyran-2,6(3H)-dione	2422	2427	54.69	-	-	-	
**Terpenoids**							
Linalool oxide	1069	1073	464.22	190	2.4	Sweet, floral, creamy	Ho et al. 2015
Camphor	1138	1143	221.52	1000	0.2	warm, minty	Čiča et al. 2022
Menthol	1150	1158	134.76	1	134.8	Menthol	Burdock 2010
Myrtenal	1182	1187	57.45	-	-	-	
Citronellol	1226	1228	34.39	40	0.8	Fresh kaffir lime leaf, citrus	Yamamoto et al. 2004
Thymol	1284	1290	28.67	188	0.1	Spicy, phenolic, medicine-like	Guo et al. 2021
Geranylacetone	1441	1447	45.00	0.48	93.7	Fresh, Floral, Rose, Green,	Fan et al. 2018
**Acids**							
Decanoic acid	1377	1382	62.81	1000	0.06	rancid fat	Delgado et al. 2022
Dodecanoic acid	1556	1562	59.10	-	-	-	

[a] Retention index based on a homologous series of normal alkanes. [b] Literature RI taken from NIST 16 or ADAMS libraries. [c] OT, odor threshold as reported in the literature. [d] OAV, Odor activity value; [e] Odor descriptor as reported in the literature; —, data not detected or not available.

References for the Supplementary Table 1:

Burdock GA. Fenaroli's handbook of flavor ingredients. CRC press, 2016.

Čiča KH; Lukin P; Derewiaka D; Mrvčić J; Stanzer D. Chemical Composition, Physical Properties, and Aroma Profile of Ethanol Macerates of Mistletoe (*Viscum album*). Beverages 2022, 8, 46. https://doi.org/10.3390/beverages8030046.

Delgado JA, Sánchez-Palomo E, Alises MO, Viñas MG. Chemical and sensory aroma typicity of La Mancha Petit Verdot wines. Lwt. 2022;162:113418.

Fan J, Zhang W, Zhou T, Zhang D, Zhang D, Zhang L, Wang G, Cao F. Discrimination of Malus Taxa with Different Scent Intensities Using Electronic Nose and Gas Chromatography⁻Mass Spectrometry. Sensors (Basel). 2018 Oct 12;18(10):3429. doi: 10.3390/s18103429.

Gemert B. V. Benzo and Naphthopyrans (Chromenes). In Organic Photochromic and Thermochromic Compounds; Crano, J. C., Guglielmetti, R., Eds.; Plenum Press: New York, 1999; Vol. 1, Chapter 3.

Guo X, Ho CT, Wan X, Zhu H, Liu Q, Wen Z. Changes of volatile compounds and odor profiles in Wuyi rock tea during processing. Food Chem. 2021 Mar 30;341(Pt 1):128230. doi: 10.1016/j.foodchem.2020.128230.

Ho CT, Zheng X, Li S. Tea aroma formation. Food Science and Human Wellness. 2015;4(1):9-27. 10.1016/j.fshw.2015.04.001.

Kreck M, Mosandl A. Synthesis, structure elucidation, and olfactometric analysis of lilac aldehyde and lilac alcohol stereoisomers. J Agric Food Chem. 2003 Apr 23;51(9):2722-6. doi: 10.1021/jf021140q.

Lasekan O. Volatile constituents of roasted tigernut oil (*Cyperus esculentus* L.). J Sci Food Agric. 2013 Mar 30;93(5):1055-61. doi: 10.1002/jsfa.5846. Epub 2012 Aug 30. PMID: 22936608.

Paravisini L, Prot A, Gouttefangeas C, Moretton C, Nigay H, Dacremont C, Guichard E. Characterisation of the volatile fraction of aromatic caramel using heart-cutting multidimensional gas chromatography. Food Chem. 2015 Jan 15;167:281-9. doi: 10.1016/j.foodchem.2014.06.101.

Pino, J.A. and Mesa, J. (2006), Contribution of volatile compounds to mango (Mangifera indica L.) aroma. Flavour Fragr. J., 21: 207-213. https://doi.org/10.1002/ffj.1703

Pino, J.A. (2012), Analysis of odour-active compounds of black mangrove (*Avicennia germinans* L.) honey by solid-phase microextraction combined with gas chromatography–mass spectrometry and gas chromatography–olfactometry. International Journal of Food Science & Technology, 47: 1688-1694. https://doi.org/10.1111/j.1365-2621.2012.03021.x

Piornos JA, Delgado A, de La Burgade RCJ, Methven L, Balagiannis DP, Koussissi E, Brouwer E, Parker JK. Orthonasal and retronasal detection thresholds of 26 aroma compounds in a model alcohol-free beer: Effect of threshold calculation method. Food Res Int. 2019 Sep;123:317-326. doi: 10.1016/j.foodres.2019.04.034.

Söllner K, Schieberle P. Decoding the key aroma compounds of a Hungarian-type salami by molecular sensory science approaches. J Agric Food Chem. 2009 May 27;57(10):4319-27. doi: 10.1021/jf900402e.

Song J, Shao Y, Yan Y, Li X, Peng J, Guo L. Characterization of volatile profiles of three colored quinoas based on GC-IMS and PCA. LWT. 2021;146:111292. doi: 10.1016/j.lwt.2021.111292.

Sun H, Ni H, Yang Y, Wu L, Cai HN, Xiao AF, Chen F. Investigation of sunlight-induced deterioration of aroma of pummelo (Citrus maxima) essential oil. J Agric Food Chem. 2014 Dec 10;62(49):11818-30. doi: 10.1021/jf504294g.

Wang Q, Zhang J, Jia W, Yu X, Chen J, Sun Y, Wei Z, Yang S, Tang X, Zeng X, Lin L. Aerobic oxidation of 5-[(formyloxy)methyl]furfural to 2,5-furandicarboxylic acid over MoCuOx catalyst. Molecular Catalysis. 2022; 517:111986. doi: 10.1016/j.mcat.2021.111986.

Yamamoto T, Shimada A, Ohmoto T, Matsuda H, Ogura M, Kanisawa T. Olfactory study on optically active citronellyl derivatives. Flavour and Fragrance Journal, 2004;19(2): 121-133.

Zhu Y., Chen X., Pan N., Liu S., Su Y., Xiao M., Shi W., Liu Z.J.L. The effects of five different drying methods on the quality of semi-dried Takifugu obscurus fillets. LWT-Food Science and Technology. 2022;161 doi: 10.1016/j.lwt.2022.113340.
